# Serum ceramides in early pregnancy as predictors of gestational diabetes

**DOI:** 10.1038/s41598-023-40224-3

**Published:** 2023-08-15

**Authors:** Sanna Mustaniemi, Elina Keikkala, Eero Kajantie, Markku Nurhonen, Antti Jylhä, Laure Morin-Papunen, Hanna Öhman, Tuija Männistö, Hannele Laivuori, Johan G. Eriksson, Reijo Laaksonen, Marja Vääräsmäki, The FinnGeDi Study Group, Risto Kaaja, Mika Gissler, Anneli Pouta

**Affiliations:** 1 Clinical Medicine Research Unit, Medical Research Center Oulu, Oulu University Hospital and University of Oulu, PL 23, 90029 Oulu, Finland; 2 Population Health Unit, Department of Public Health and Welfare, Finnish Institute for Health and Welfare, Helsinki, Oulu, Finland; 3 Children’s Hospital, University of Helsinki and Helsinki University Hospital, Helsinki, Finland; 4 Department of Clinical and Molecular Medicine, Norwegian University of Science and Technology, Trondheim, Norway; 5 Zora Biosciences Oy, Espoo, Finland; 6 Biobank Borealis of Northern Finland, Oulu University Hospital, Oulu, Finland; 7 Faculty of Medicine, University of Oulu, Oulu, Finland; 8 Nordlab, Oulu, Finland; 9 Department of Obstetrics and Gynecology, Center for Child, Adolescence and Maternal Health, Tampere University Hospital and Faculty of Medicine and Health Technology, Tampere University, Tampere, Finland; 10 Medical and Clinical Genetics, University of Helsinki and Helsinki University Hospital, Helsinki, Finland; 11 Institute for Molecular Medicine Finland, Helsinki Institute of Life Science, University of Helsinki, Helsinki, Finland; 12 Department of General Practice and Primary Health Care, University of Helsinki and Helsinki University Hospital, Helsinki, Finland; 13 Folkhälsan Research Center, Helsinki, Finland; 14 Department of Obstetrics and Gynecology and Human Potential Translational Research Programme, Yong Loo Lin School of Medicine, National University of Singapore, Singapore, Singapore; 15 Singapore Institute for Clinical Sciences (SICS), Agency for Science, Technology, and Research, Singapore, Singapore; 16 Institute of Clinical Medicine, Internal Medicine, Turku University Hospital, University of Turku, Turku, Finland; 17 Department of Information Services, Finnish Institute for Health and Welfare, Helsinki, Finland; 18 Region Stockholm and Department of Molecular Medicine and Surgery, Academic Primary Health Care Centre, Karolinska Institute, Stockholm, Sweden; 19 Department of Government Services, Finnish Institute for Health and Welfare, Helsinki, Finland

**Keywords:** Predictive markers, Diabetes, Dyslipidaemias, Obesity, Pre-diabetes

## Abstract

Ceramides contribute to the development of type 2 diabetes but it is uncertain whether they predict gestational diabetes (GDM). In this multicentre case–control study including 1040 women with GDM and 958 non-diabetic controls, early pregnancy (mean 10.7 gestational weeks) concentrations of four ceramides—Cer(d18:1/16:0), Cer(d18:1/18:0), Cer(d18:1/24:0) and Cer(d18:1/24:1)—were determined by a validated mass-spectrometric method from biobanked serum samples. Traditional lipids including total cholesterol, LDL, HDL and triglycerides were measured. Logistic and linear regression and the LASSO logistic regression were used to analyse lipids and clinical risk factors in the prediction of GDM. The concentrations of four targeted ceramides and total cholesterol, LDL and triglycerides were higher and HDL was lower among women with subsequent GDM than among controls. After adjustments, Cer(d18:1/24:0), triglycerides and LDL were independent predictors of GDM, women in their highest quartile had 1.44-fold (95% CI 1.07–1.95), 2.17-fold (95% CI 1.57–3.00) and 1.63-fold (95% CI 1.19–2.24) odds for GDM when compared to their lowest quartiles, respectively. In the LASSO regression modelling ceramides did not appear to markedly improve the predictive performance for GDM alongside with clinical risk factors and triglycerides. However, their adverse alterations highlight the extent of metabolic disturbances involved in GDM.

## Introduction

Gestational diabetes (GDM) is one of the most common pregnancy complications, affecting between 7 and 28% of pregnancies worldwide^1^. GDM predisposes both the woman and her child to major short- and long-term adverse health outcomes^2–10^. Among women with a history of GDM, up to half develop type 2 diabetes later in life^4,6^ and they also have a higher prevalence of several cardiometabolic risks, including hypertension, dyslipidaemia, metabolic syndrome and cardiovascular disease^9–11^.

Physiological insulin resistance and alterations in lipid metabolism characterize a normal pregnancy^12,13^. Among women with GDM, chronic insulin resistance is often present before conception, and hyperglycaemia develops when pancreatic compensatory mechanisms fail^12,14^. Besides being linked to hyperglycaemia, insulin resistance is also linked to alterations in lipid metabolism during pregnancy^15^. It is well documented that circulating traditional lipids, especially triglycerides, are associated with an increased risk of GDM^15,16^. However, the relationships between lipid metabolism and diabetic pathways are far more complex and require a better understanding than can be gained from measuring traditional lipids^17^.

Ceramides (Cer) are sphingolipids which have a key role in the development of insulin resistance, type 2 diabetes and cardiovascular disease^17–22^. Ceramides, the precursors of more complex sphingolipids, are formed from a sphingoid base attached to a fatty acid of varying length ranging from 14 to 34 carbon (C) atoms^20^. In particular, the oversupply of saturated fat leads to the accumulation of these biologically active metabolites in several tissues, such as the liver, adipose tissue and skeletal muscle. Ceramides can modify several intracellular signalling pathways, such as inhibiting insulin signalling by blocking protein kinase B (Akt), leading to decreased insulin-induced glucose uptake^17,20^.

Circulating ceramides are already elevated years before the onset of type 2 diabetes^23^. The value of three ceramides—Cer(d18:1/16:0), Cer(d18:1/18:0) and Cer(d18:1/24:1)—and their ratios to Cer(d18:1/24:0) in predicting cardiovascular risk, especially cardiovascular death has been previously demonstrated and validated for clinical use^21,22,24^. Further, ceramide species which contain C16:0 (palmitic acid) and C18:0 (stearic acid) have showed the strongest association with insulin resistance and incident type 2 diabetes^17,18,23,25^ and the ratio of ceramide stearic to palmitic acid [Cer(d18:1/18:0)/Cer(d18:1/16:0) ratio] has found to be an independent predictor for incident type 2 diabetes^18^. Although ceramides and the Cer(d18:1/18:0)/Cer(d18:1/16:0) ratio can be used to identify nonpregnant individuals at the greatest risk for type 2 diabetes, it is not clear whether they are useful in the prediction of GDM. In three studies, higher concentrations of early-pregnancy ceramide species containing C14:0^26^, C18:1^27,28^, and C18:0^28^ were associated with subsequent GDM.

The aim of this large, case–control study to examine whether four previously validated^24^ type 2 diabetes- and cardiovascular disease-associated serum ceramides Cer(d18:1/16:0), Cer(d18:1/18:0), Cer(d18:1/24:0) and Cer(d18:1/24:1) and the Cer(d18:1/18:0)/Cer(d18:1/16:0) ratio, as well as traditional lipids, measured in early pregnancy, are predictors of subsequent GDM.

## Methods

### Study population and design

This case–control study is a part of the clinical genetic arm of the Finnish Gestational Diabetes (FinnGeDi) study, which has been described in detail^29,30^. Briefly, 1146 women with GDM and 1066 pregnant controls with no diabetes were recruited between 1 February 2009 and 31 December 2012 from delivery units in seven Finnish delivery hospitals, each serving its own geographic catchment area. Women with GDM were recruited at the delivery units as they entered to give birth, and the next-consenting mother with no diabetes giving birth at the same unit was recruited as a control. Women with multiple pregnancies or pregestational diabetes were excluded.

According to the Finnish National Current Care Guidelines, comprehensive screening of GDM by a 2 h 75 g oral glucose tolerance test (OGTT) was performed on all women at the 24th–28th weeks of gestation except for those with a very low risk for GDM (normal-weight primiparous women under 25 years without a family history of type 2 diabetes and normal-weight multiparous women under 40 years without a history of GDM and macrosomic births)^31^. OGTT was performed already at the 12th–16th weeks of gestation on high-risk women (history of GDM, BMI > 35 kg/m^2^, glucosuria in early pregnancy, type 2 diabetes in a first-degree relative, systemic corticosteroid therapy or polycystic ovary syndrome), and it was repeated at the 24th–28th weeks of gestation if the first OGTT was normal. The cut-off values for the venous glucose concentrations were as follows: fasting ≥ 5.3 mmol/L; 1 h ≥ 10.0 mmol/L; and 2 h ≥ 8.6 mmol/L. At least one abnormal value was diagnostic for GDM. The GDM status of each participant was confirmed from the medical records.

### Clinical data

Participants completed background questionnaires about their lifestyles and medical and family histories. Detailed data on pregnancy and delivery were collected from the hospital and maternal welfare clinic records and combined with individually linked register data obtained from the Finnish Medical Birth Register (FMBR).

Data on maternal age at delivery, parity and smoking during pregnancy were obtained from the FMBR. Self-reported maternal height and pre-pregnancy weight were obtained from the maternal welfare clinic records, and BMI (kg/m^2^) was calculated. Gestational weight gain was calculated as the difference between the pre-pregnancy weight and the weight at the last antenatal visit (≥ 35 weeks of gestation). Based on the questionnaire data, educational attainment was categorised as basic or less, upper secondary, lower-level tertiary or upper-level tertiary. Chronic hypertension was defined as systolic blood pressure ≥ 140 mmHg or diastolic blood pressure ≥ 90 mmHg measured repeatedly or the use of antihypertensive medication before 20 weeks of gestation, while gestational hypertension was defined when hypertension appeared after 20 weeks of gestation. Pre-eclampsia was considered when hypertension appeared after 20 weeks of gestation and was accompanied with proteinuria (≥ 300 mg/day or two ≥ 1 + readings on a dipstick test). Data on previous pregnancies, including prior GDM, were obtained from the questionnaire and the FMBR. The participants’ family history of type 2 diabetes was taken from the questionnaire.

### Serum samples and laboratory analysis

The maternal early pregnancy serum samples were obtained via the Finnish Maternity Cohort (FMC), a nationwide biobank containing leftover serum samples from routine the early pregnancy routine infectious disease screening. Therefore, fasting before sampling was not required. The samples were stored at − 25 °C in the Biobank Borealis of Northern Finland.

**Figure 1:**
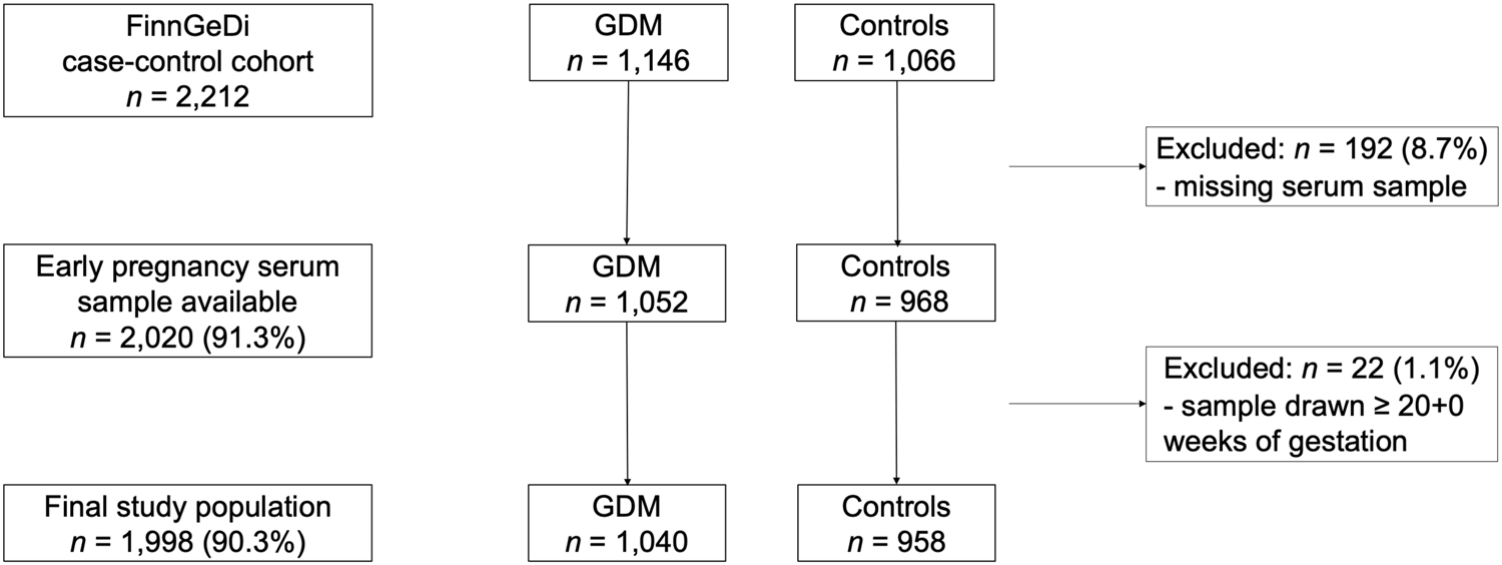
Flowchart of the study population. *FinnGeDi* Finnish Gestational Diabetes study, *GDM* gestational diabetes.

The number of analysed samples was 2020 (91.3%). The background characteristics of those participants with missing samples (total *n* = 192: *n* = 124, no sample in the biobank; *n* = 68, sample was depleted) did not significantly differ from those of the samples included. Samples drawn after 20 weeks of pregnancy (*n* = 22) were excluded. The samples were drawn, on average, at 10.7 (SD 2.1) weeks of gestation. Finally, 1998 participants (1040 with GDM and 958 controls) were included in the analyses (Fig. 1). All laboratory analyses were performed blinded to the GDM status of the participants and all other phenotypic data.

Four ceramide lipids—Cer(d18:1/16:0), Cer(d18:1/18:0), Cer(d18:1/24:0), Cer(d18:1/24:1)—were measured using a validated, targeted, liquid chromatography–tandem mass spectrometry assay by Zora Biosciences Oy in Espoo, Finland^19,24^. Detailed laboratory methods have been described previously^24^. Briefly, 10 µl serum samples were spiked with ^2^H (deuterium [D])-labelled internal standards—D7-Cer(d18:1/16:0), D7-Cer(d18:1/18:0), D7-Cer(d18:1/24:0) and D7-Cer(d18:1/24:1)—and extracted in isopropanol:ethyl acetate (8:2, vol./vol.). Quantification of the individual ceramides was performed in multiple-reaction-monitoring mode and assessed through calibration line samples composed of known amounts of synthetic Cer(d18:1/16:0), Cer(d18:1/18:0), Cer(d18:1/24:0) and Cer(d18:1/24:1) and corresponding ^2^H-labelled standards. The peak area ratio for each ceramide to its corresponding ^2^H-labelled form was calculated and plotted against the added concentration of ceramide, followed by linear regression analysis. Concentrations of ceramides are presented in µmol/L. The ratio of Cer(d18:1/18:0)/Cer(d18:1/16:0) was calculated. The NordLab clinical laboratory analysed traditional lipids (total cholesterol, low-density lipoprotein [LDL], high-density lipoprotein [HDL] and triglycerides) using a standard enzymatic assay on a Siemens Advia automatic biochemical analyser (Siemens Healthineers, Germany) in Oulu, Finland. Random first-trimester samples of pooled serum from the FMC were incorporated with the sample runs; these samples acted as internal controls, and the inter-assay coefficients of variation (CVs, SD/mean) were derived from these samples and intra-assay CVs were derived from the intra-assay quality control samples (Supplemental Table 1).

### Statistics

Statistical analyses were performed using SPSS 28.0 and R software (version 4.2.1). The baseline characteristics of the study participants were described using the unpaired Student’s *t*-test for continuous variables (expressed as means and standard deviations, SDs) and the χ^2^ test for categorical variables (expressed as frequencies). The main outcome was GDM. We compared the means of traditional lipids, ceramides and the Cer(d18:1/18:0)/Cer(d18:1/16:0) ratio using both linear and logistic regression. To estimate the association of each variable with GDM, mean differences and odds ratios (ORs) with 95% confidence intervals (CI) per SD and per quartile (Q2–Q4) were calculated using the lowest quartile (Q1) as a reference. Model 1 was unadjusted. In Model 2, the results were adjusted for pre-pregnancy BMI, age, parity (dichotomous variable: primipara/multipara) and gestational weeks at sampling. Model 3 was adjusted for Model 2 and educational attainment, a history of GDM, parental type 2 diabetes and delivery unit. Categorical variables were added as dummy-coded, with a separate dummy variable indicating missing values. The directed acyclic graph summarising the hypothetical causality between ceramides and traditional lipids and GDM, and potential confounding variables used in the regression analyses is shown in Supplemental Fig. 1.

Last, to assess the relative contributions of covariates on the risk of GDM, a least absolute shrinkage and selection operator (LASSO) logistic regression analysis was constructed (using the R package ‘*glmnet*’); this regularization method is useful in selecting parsimonious predictive models, particularly when there is multicollinearity among covariates (as is the case with lipids in this study)^32^. Models with different sets of covariates were considered. First, clinical predictors for GDM (pre-pregnancy BMI, age, parity, a history of GDM, parental type 2 diabetes), educational attainment and delivery unit, altogether 16 covariates, were considered. Then, all four ceramides, the Cer(d18:1/18:0)/Cer(d18:1/16:0) ratio and traditional lipids (altogether nine covariates) were included. Furthermore, models including logarithmic, square, cubic and square root transformations of continuous covariates were considered.

The LASSO algorithm shrinks the regression coefficients using a regularization parameter lambda. As lambda increases, the coefficients of covariates deemed less important tend towards zero. The model corresponding to the level of regularization with optimal predictive performance was selected. To achieve this, for a range of values of lambda, tenfold cross validation was repeated 100 times the average area under the curve (AUC) value, and the average root mean squared error (RMSE) was recorded. The model corresponding to the optimal value (largest AUC or smallest RMSE) of lambda was selected. Furthermore, tenfold cross-validation was also applied to assess the out-of-sample prediction accuracy of various models. Here, the model was repeatedly fitted using 90% of the data, and the accuracy of the model predictions to the actual observed values (according to the AUC or RMSE criteria) for the remaining 10% of the data not used in the model fitting (holdout data) was evaluated.

### Power analysis

The power of the study was sufficient to identify small differences between the study groups. With a power of 0.80, a significance level of 0.05 and an effect size of d 0.13, we were able to detect a difference of 0.13 SD in lipids between women with GDM and the controls.

### Ethical aspects

The study was carried out according to the Declaration of Helsinki and approved by the Ethics Committee of Northern Ostrobothnia Hospital District (Reference Number 33/2008), the Finnish Institute for Health and Welfare and the scientific committee of the Northern Finland Biobank Borealis. All participants gave written informed consent after full explanation of the purpose and nature of all procedures used.

## Results

### Clinical characteristics

The women with GDM were older, less often primiparous and had higher BMI and blood pressure than the controls (Table 1). In the GDM group, 41.2% of multiparous women had a history of GDM, compared with 5.6% in the control group. Of the women with GDM, 195 (19.1%) received antidiabetic medication, including 182 (17.9%) receiving insulin and 22 (2.2%) receiving metformin. A family history of type 2 diabetes was more common in the GDM group than in the control group, 30.5% and 17.5%, respectively.

**Table 1:** Baseline characteristics of study participants (*n* = 1998).

Baseline characteristic	GDM (*n* = 1040)		Controls (*n* = 958)		*p* value^a^
	Mean (SD)/*n* (%)	No. of missing	Mean (SD)/*n* (%)	No. of missing	
Age at delivery, years	32.1 (5.4)	0	29.5 (5.2)	0	< 0.001
Primiparity, n (%)	445 (42.8%)	0	456 (47.6%)	0	0.031
Prepregnancy weight, kg	76.5 (17.0)	1	64.6 (12.2)	0	< 0.001
Height, cm	164.9 (5.9)	0	165.4 (5.9)	0	0.031
Pre-pregnancy BMI, kg/m^2^	28.1 (6.0)	1	23.6 (4.1)	0	< 0.001
Gestational weight gain, kg	12.3 (5.8)	83	14.8 (5.0)	30	< 0.001
Educational attainment		104		116	0.013
Basic or less, *n* (%)	63 (6.7%)		36 (4.3%)		
Upper secondary, *n* (%)	439 (46.9%)		385 (45.7%)		
Lower-level tertiary, *n* (%)	245 (26.2%)		208 (24.7%)		
Upper-level tertiary, *n* (%)	189 (20.2%)		213 (25.3%)		
Smoking during pregnancy, *n* (%)	168 (16.2%)	3	142 (14.8%)	1	0.402
Chronic hypertension, *n* (%)	168 (16.2%)	1	43 (4.5%)	0	< 0.001
Gestational hypertension, *n* (%)	214 (20.6%)	1	140 (14.6%)	0	< 0.001
Pre-eclampsia, *n* (%)	61 (5.9%)	1	24 (2.5%)	0	< 0.001
Antidiabetic medication *n* (%)	195 (19.1%)	21	0	0	
Insulin, *n* (%)	182 (17.9%)	21	0	0	
Metformin, *n* (%)	22 (2.2%)	28	0	0	
Prior GDM among multiparous women, *n* (%)	245 (41.2%)	0	28 (5.6%)	0	< 0.001
Participant’s mother’s GDM	80 (9.0%)	151	21 (2.7%)	170	< 0.001
Family history of type 2 diabetes	279 (30.5%)	125	143 (17.5%)	143	< 0.001
Mother’s type 2 diabetes	139 (15.3%)	133	55 (6.8%)	149	< 0.001
Father’s type 2 diabetes	174 (19.7%)	156	98 (12.1%)	149	< 0.001

*GDM* gestational diabetes.

^a^*p* values based on the Student’s *t* test or χ^2^ test.

### Ceramides

Overall, the early pregnancy concentrations of all four ceramides—Cer(d18:1/16:0), Cer(d18:1/18:0), Cer(d18:1/24:0) and Cer(d18:1/24:1)—and the Cer(d18:1/18:0)/Cer(d18:1/16:0) ratio were higher among women with GDM compared to women without GDM (Model 1) (Tables 2 and 3). After considering pre-pregnancy BMI, maternal age, parity and gestational weeks at sampling (Model 2), Cer(d18:1/18:0), Cer(d18:1/24:0) and Cer(d18:1/24:1) predicted GDM. After further adjustments in Model 3, including history of GDM and parental type 2 diabetes, Cer (d18:1/24:0) was an independent predictor for GDM.

**Table 2:** Means (SD) and mean differences (95% CI) of ceramides and traditional lipids in early pregnancy in women with subsequent gestational diabetes (GDM) compared with non-diabetic women (*n* = 1998).

	GDM (*n* = 1040)	Controls (*n* = 958)	Model 1		Model 2		Model 3	
	Mean (SD)	Mean (SD)	Mean difference (95% CI)	*p* value	Mean difference (95% CI)	*p* value	Mean difference (95% CI)	*p* value
Ceramides								
Cer(d18:1/16:0), µmol/l	0.306 (0.078)	0.297 (0.073)	0.009 (0.003 to 0.016)	0.006	0.004 (− 0.003 to 0.012)	0.293	0.002 (− 0.006 to 0.009)	0.649
Cer(d18:1/18:0), µmol/l	0.099 (0.039)	0.086 (0.031)	0.013 (0.010 to 0.016)	< 0.001	0.004 (0.000 to 0.007)	0.029	0.001 (− 0.002 to 0.005)	0.401
Cer(d18:1/24:0), µmol/l	2.119 (0.587)	1.969 (0.506)	0.150 (0.120 to 0.198)	< 0.001	0.099 (0.046 to 0.152)	< 0.001	0.076 (0.022 to 0.130)	0.006
Cer(d18:1/24:1), µmol/l	1.308 (0.346)	1.211 (0.302)	0.097 (0.069 to 0.126)	< 0.001	0.038 (0.007 to 0.069)	0.015	0.020 (− 0.011 to 0.052)	0.207
Cer(d18:1/18:0)/Cer(d18:1/16:0) ratio	0.326 (0.111)	0.292 (0.085)	0.034 (0.026 to 0.043)	< 0.001	0.009 (0.000 to 0.019)	0.050	0.005 (− 0.005 to 0.015)	0.312
Traditional lipids								
Cholesterol, mmol/l	4.81 (0.80)	4.63 (0.80)	0.18 (0.11 to 0.25)	< 0.001	0.09 (0.02 to 0.17)	0.014	0.08 (0.00 to 0.15)	0.056
LDL, mmol/l	2.54 (0.68)	2.29 (0.68)	0.25 (0.19 to 0.31)	< 0.001	0.14 (0.07 to 0.20)	< 0.001	0.11 (0.04 to 0.17)	< 0.001
HDL, mmol/l	0.82 (0.17)	0.86 (0.18)	− 0.04 (− 0.06 to − 0.03	< 0.001	− 0.02 (− 0.04 to − 0.01)	0.006	− 0.02 (− 0.03 to 0.00)	0.061
Triglycerides, mmol/l	1.70 (0.87)	1.36 (0.53)	0.34 (0.28 to 0.40)	< 0.001	0.21 (0.14 to 0.27)	< 0.001	0.19 (0.12 to 0.26)	< 0.001

*Cer* ceramide, *HDL* high-density lipoprotein, *LDL* low-density lipoprotein.

Model 1: Linear regression, unadjusted.

Model 2: Linear regression adjusted for pre-pregnancy BMI, maternal age, parity and gestational weeks at sampling.

Model 3: Linear regression adjusted for Model 2 and education, history of GDM, parental type 2 diabetes and delivery unit.

**Table 3:** Odds ratios (ORs) per standard deviation (SD) for gestational diabetes (GDM) (*n* = 1998).

	OR (95% CI)	OR (95% CI)	OR (95% CI)
	Model 1	Model 2	Model 3
Ceramides			
Cer(d18:1/16:0), µmol/l	1.14 (1.04–1.25)	1.07 (0.96–1.18)	1.03 (0.92–1.15)
Cer(d18:1/18:0), µmol/l	1.48 (1.34–1.63)	1.14 (1.01–1.27)	1.06 (0.94–1.20)
Cer(d18:1/24:0), µmol/l	1.33 (1.21–1.47)	1.19 (1.07–1.33)	1.15 (1.03–1.29)
Cer(d18:1/24:1), µmol/l	1.38 (1.25–1.51)	1.13 (1.02–1.26)	1.07 (0.95–1.20)
Cer(d18:1/18:0)/Cer(d18:1/16:0) ratio	1.44 (1.31–1.59)	1.12 (1.00–1.25)	1.07 (0.95–1.20)
Traditional lipids			
Cholesterol, mmol/l	1.26 (1.15–1.38)	1.11 (1.00–1.24)	1.09 (0.97–1.21)
LDL, mmol/l	1.47 (1.34–1.62)	1.23 (1.10–1.37)	1.17 (1.04–1.31)
HDL, mmol/l	0.79 (0.73–0.87)	0.87 (0.79–0.97)	0.90 (0.81–1.01)
Triglycerides, mmol/l	1.94 (1.71–2.20)	1.48 (1.29–1.70)	1.43 (1.24–1.65)

*Cer* ceramide, *HDL* high-density lipoprotein, *LDL* low-density lipoprotein.

Model 1: Linear regression, unadjusted.

Model 2: Linear regression adjusted for pre-pregnancy BMI, maternal age, parity and gestational weeks at sampling.

Model 3: Linear regression adjusted for Model 2 and education, history of GDM, parental type 2 diabetes and delivery unit.

**Figure 2:**
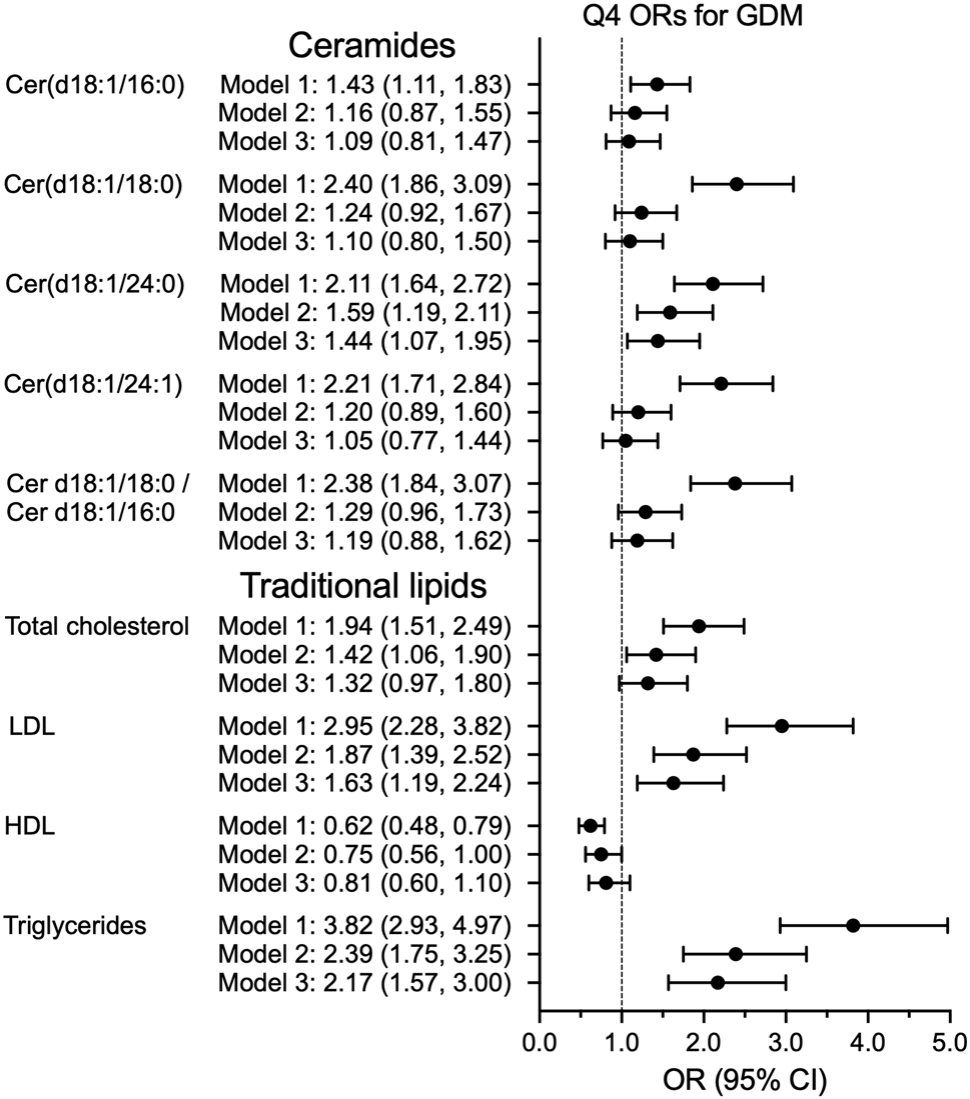
Odds ratios (ORs) per 4th quartile (Q4) for GDM. Model 1 was unadjusted. Model 2 was adjusted with pre-pregnancy BMI, age, parity (dichotomous variable) and gestational weeks at sampling. Model 3 was adjusted for Model 2 and educational attainment, history of GDM, parental type 2 diabetes and delivery unit. *Cer* ceramide, *GDM* gestational diabetes, *HDL* high-density lipoprotein, *LDL* low-density lipoprotein.

When ORs per quartiles were assessed for GDM and the highest quartile was compared with the lowest quartile, in unadjusted Model 1, Cer(d18:1/16:0) showed 1.43-fold odds (95% CI 1.11–1.83), Cer(d18:1/18:0) showed 2.40-fold odds (95% CI 1.86–3.09), Cer(d18:1/24:0) showed 2.11-fold odds (95% CI 1.64–2.72), Cer(d18:1/24:1) showed 2.21-fold odds (95% CI 1.71–2.84) and the Cer(d18:1/18:0)/Cer(d18:1/16:0) ratio showed 2.38-fold odds (95% CI 1.84–3.07) for GDM (Fig. 2, Supplemental Table 2). After further adjustments (Model 3), Cer(d18:1/24:0) was an independent predictor of GDM (OR 1.44, 95% CI 1.07–1.95). In a comparison of the quartiles, the other ceramides and the Cer(d18:1/18:0)/Cer(d18:1/16:0) ratio were not significant after adjustments.

### Traditional lipids

Higher concentrations of LDL and triglycerides in early pregnancy were independent predictors for GDM (Tables 2 and 3). After adjustments (Model 3), the women in the highest triglyceride quartile (1.82–13.0 mmol/L) had 2.17-fold odds (95% CI 1.57–3.00) for GDM compared with those in the lowest quartile (0.50–1.06 mmol/L) (Fig. 2, Supplemental Table 2). In addition, the women in the highest quartile of LDL (2.83–5.64 mmol/L) had 1.63-fold odds (95% CI 1.19–2.24) for GDM, compared with the lowest quartile (0.72–1.92 mmol/L).

### Lipids selected by the LASSO regression model

**Figure 3:**
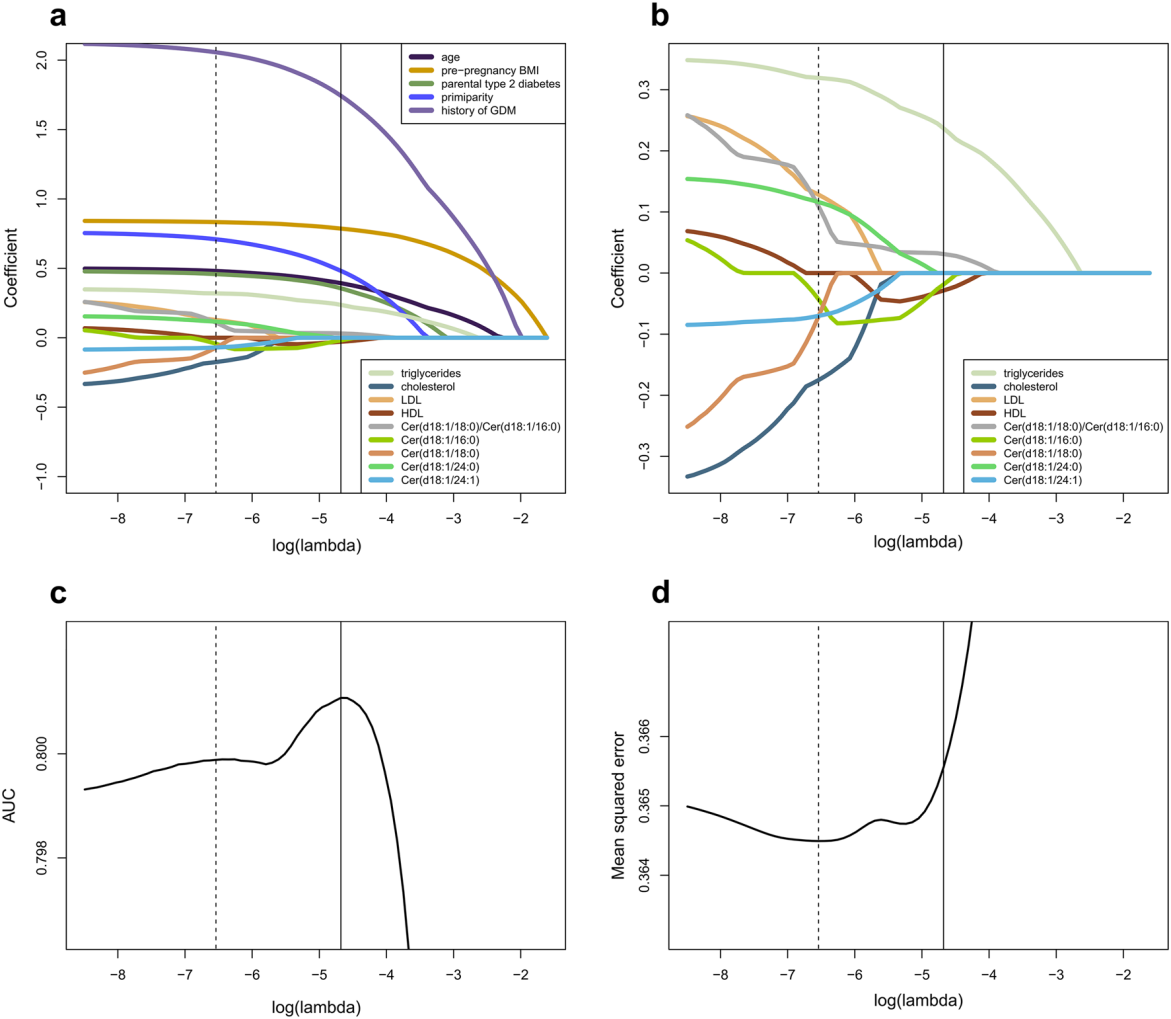
Selection of LASSO model. In each plot (a-d), an optimal predictive model is indicated by a vertical continuous line for the highest area under the curve (AUC) value and by a dashed vertical line for the lowest root mean squared error (RMSE). For a range of values of lambda, tenfold cross-validation was repeated 100 times, and the average of these values are plotted. (**a**) The coefficients of the clinical risk factors, ceramides, Cer(d18:1/18:0)/Cer(d18:1/16:0) ratio and traditional lipids in the LASSO regression by the magnitude of log(lambda). Delivery unit and educational attainment not shown. (**b**) The coefficients of the ceramides, Cer(d18:1/18:0)/Cer(d18:1/16:0) ratio and traditional lipids in the LASSO regression by the magnitude of log(lambda). (**c**) Selection for the optimal predictive model with the highest AUC (continuous line). Triglycerides, Cer(d18:1/18:0)/Cer(d18:1/16:0) ratio, Cer(d18:1/16:0) and HDL with nonzero coefficients were selected. (**d**) Selection for the optimal predictive model with lowest RMSE (dashed line). Triglycerides, LDL, Cer(d18:1/24:0), the Cer(d18:1/18:0)/Cer(d18:1/16:0) ratio, Cer(d18:1/16:0), Cer(d18:1/18:0), Cer(d18:1/24:1) and total cholesterol with nonzero coefficients were selected. *Cer* ceramide, *HDL* high-density lipoprotein, *LASSO* Least absolute shrinkage and selection operator, *LDL* low-density lipoprotein.

When the clinical predictors for GDM were included, ceramides and traditional lipids proved to possess limited importance in improving the predictive power of the regression equations (Fig. 3). Nevertheless, a set of variables consisting of triglycerides, other traditional lipids and a subset consisting of one or more of the ceramides or the Cer(d18:1/18:0)/Cer(d18:1/16:0) ratio were consistently included in the selected LASSO regression equation. When variable selection was performed using the highest AUC criteria, triglycerides, the Cer(d18:1/18:0)/Cer(d18:1/16:0) ratio, Cer(d18:1/16:0) and HDL were selected for the model (Fig. 3). While seeking the lowest RMSE, triglycerides, LDL, Cer(d18:1/24:0), the Cer(d18:1/18:0)/Cer(d18:1/16:0) ratio, Cer(d18:1/16:0), Cer(d18:1/18:0), Cer(d18:1/24:1) and total cholesterol were selected. These two optimal LASSO models do not practically differ in their predictive performance, and there is very little change in the predictive performance of the regression models across a relatively wide range of the regularization parameter values. Using transformations of continuous variables did not improve the predictive power.

In the out-of-sample prediction of GDM, the AUC value was 0.796 for the clinical risk factors (Supplemental Fig. 2). The combination of clinical risk factors and triglycerides and/or other traditional lipids increased the AUC to 0.801. Finally, adding four ceramides and the Cer(d18:1/18:0)/Cer(d18:1/16:0) ratio with clinical risk factors, traditional lipids resulted in a similar AUC of 0.801. The corresponding out-of-sample RMSEs were 0.430, 0.427, and 0.427.

## Discussion

This case–control study including 1040 women who developed GDM and 958 pregnant controls demonstrated that the early pregnancy serum concentration of ceramides Cer(d18:1/16:0), Cer(d18:1/18:0), Cer(d18:1/24:0), Cer(d18:1/24:1) and the Cer(d18:1/18:0)/Cer(d18:1/16:0) ratio, as well as triglycerides, LDL and total cholesterol were higher and HDL was lower among women who subsequently developed GDM. In logistic regression models with a single predictor, Cer(d18:1/24:0), triglycerides and LDL were independent predictors for GDM. In the LASSO regression modelling, in addition to clinical risk factors and triglycerides ceramides did not appear to markedly improve the predictive performance for GDM.

Ceramides play a lipotoxic role in the development of insulin resistance, type 2 diabetes and cardiovascular disease^17–22^. Hypercaloric diet and obesity lead to excess delivery of fatty acids, which causes dysregulation of multiple lipid metabolic pathways and accumulation of numerous lipid subtypes such as ceramides^17,20^. Further, these changes in lipid metabolism promote insulin resistance, mitochondrial dysfunction, oxidative stress and inflammation^17^. Concentrations of several circulating ceramides elevate years before the onset of type 2 diabetes^23,33^.

Although the underlying mechanisms are not fully known, the length of the acyl chain of ceramide seems to play a role in the development of insulin resistance^17,20^. In nonpregnant populations, elevated levels of ceramides containing long acyl chains, such as C16:0 and C18:0, have shown the strongest association with insulin resistance and incident type 2 diabetes^17,18,20,23,25^. Instead, very long chains containing ceramides, such as C24:0, have been suggested as neutral or protective^17,20^; however, some studies have reported them to be associated with insulin resistance and type 2 diabetes^23,33–35^. When normoglycaemic women were studied 12 weeks after GDM pregnancy, the levels of C22:0 and C24:0 ceramide species were higher among those who develop type 2 diabetes in the long term^33^.

During pregnancy, the serum concentrations of several ceramides, as well as traditional lipids, are known to increase^13,27,36–38^. Maternal hyperlipidaemia is primarily aimed at securing fetal growth and development, especially in the third trimester^38^. Only a few studies have examined the associations of early pregnancy serum ceramide concentrations in subsequent GDM, with conflicting results^26–28,36,39,40^. Three previous studies, a prospective lipidomic study including 492 women with GDM^26^, a prospective cohort study including 53 women with GDM^27^ and a nested case–control study including 243^28^ women with GDM, reported that higher levels of circulating C14:0^26^, C18:0^27,28^, and C18:1^28^ ceramide species in early pregnancy were associated with subsequent GDM.

In line with previous findings^27,28^, we also found that the early pregnancy concentrations of Cer(d18:1/18:0) were higher in women who developed GDM compared with those who did not, but the difference was mostly explained by their higher BMI and age. Further, the difference was attenuated by other clinical risk factors for GDM, such as a history of GDM and a family history of type 2 diabetes. Although Cer(d18:1/18:0) was selected for the LASSO model, it did not improve predictive performance alongside clinical risk factors and/or triglycerides. In line with our findings, in a recent lipidomic study of 336 women with GDM, C18:0 ceramide was not independently associated with GDM^39^. Instead, they detected by the LASSO regression 10 lipid biomarkers in three categories of lipid classes, including one upregulated glycerolipid, five glycerophospholipids and four downregulated sphingolipids. Furthermore, in two lipidomics studies of 107^40^ and 100^36^ women with GDM, some di- and triacylglycerides were independent biomarkers for GDM, but ceramides were not^36,40^.

Although the Cer(d18:1/18:0)/Cer(d18:1/16:0) ratio has been shown to be an independent predictor of type 2 diabetes^18^, in this study among pregnant women, it did not improve the predictive performance of GDM when clinical risk factors, especially pre-pregnancy BMI or triglycerides, were considered. Further, we found Cer(d18:1/24:0) to be an independent predictor for GDM; in contrast, previous studies with smaller sample sizes found either an inverse association^28,41^ or no association between C24:0 and GDM^27^. Although Cer(d18:1/24:0) was positively related to GDM, it did not improve the prediction of GDM when clinical risk factors and triglycerides were considered.

Possible explanations for discordant results may be differences in sample size and settings of studies, diagnostic criteria of GDM, varying methods of determining ceramides and differences in study populations and analysing methods, including adjustments for covariates, especially where pre-pregnancy BMI plays an important role.

This study has several strengths. Four ceramides, previously validated in nonpregnant populations^19,24^, were measured during early pregnancy in a large group of pregnant women in this well-defined case–control setting. This was the first study assessing the early pregnancy levels of these ceramides and the Cer(d18:1/18:0)/Cer(d18:1/16:0) ratio together with traditional lipids and evaluating their roles as predictors for subsequent GDM. The LASSO regression, also previously applied in several lipidomic studies assessing circulating lipids in early pregnancy with subsequent GDM^26,39,42^, was selected as an efficient method to create a parsimonious predictive model in the presence of multicollinear predictors. The GDM status of each participant was confirmed from the medical records, and several potential confounders were considered in the analyses. The study provides reference data for ceramide lipids among pregnant women in relation to GDM status.

The study also has some limitations. Firstly, serum samples were taken at non-fasting state, which may have a minor effect on triglyceride levels^43^. Secondly, the majority of the participants were of Finnish ancestry, which may limit the generalisability of the findings. Thirdly, the quantifications of other ceramides, diacyl- or triacylglycerols, or lipidomic analyses could have brought a broader perspective to this subject but they were not possible to realise within this study. Finally, the results could not be validated in an external cohort; to control this limitation, the LASSO regression and cross-validation were performed.

Future studies with independent external validation are needed to confirm the findings of this study. Further, it would be important to study whether these early pregnancy alterations in lipid metabolism are related to the long-term metabolic health and development of type 2 diabetes, and whether the ceramide profile varies depending on the stage of the diabetic cascade.

The early pregnancy levels of ceramides and traditional lipids were higher among women who developed GDM compared to those who did not. Cer(d18:1/24:0), triglycerides and LDL were found to be independent predictors of GDM. Clinical risk factors played a dominant role in predicting GDM and after combined with triglycerides, ceramides did not markedly improve the predictive performance for GDM. However, adverse alterations in lipids reflects the clustering of metabolic risk factors related to GDM.

### Supplementary Information

Supplementary Information.

## Data Availability

The datasets generated and analysed during the current study are not readily available because individual-level sensitive health data cannot be shared for legal and ethical reasons. Data from the Finnish Institute for Health and Welfare can only be used for the purpose stated in the licence granted, scientific research on society by the licence applicant, and can therefore not be shared with third parties. Researchers can apply for data through the authorisation application process at the Finnish Institute for Health and Welfare. Requests to access the datasets should be directed to Sanna Mustaniemi.
