# Supplementary material for: Serum ceramides in early pregnancy as predictors of gestational diabetes
Source: Sci Rep. 2023 Aug 15;13:13274. doi: 10.1038/s41598-023-40224-3 (PMC10427660; doi:10.1038/s41598-023-40224-3)
Supplement: Supplementary file 1 — Supplementary Information. [file 41598_2023_40224_MOESM1_ESM.doc]

**Consortium**

**The FinnGeDi Study Group**

Marja Vääräsmäki^1,2^, Eero Kajantie^1,2,3,4^, Hannele Laivuori^9,10,11^, Johan G Eriksson^12,13,14,15^, Risto Kaaja^16^, Mika Gissler^17,18^, Anneli Pouta^19^

^1^Clinical Medicine Research Unit, Medical Research Center Oulu, Oulu University Hospital and University of Oulu, Oulu, Finland

^2^Population Health Unit, Department of Public Health and Welfare, Finnish Institute for Health and Welfare, Helsinki and Oulu, Finland

^3^Children’s Hospital, University of Helsinki and Helsinki University Hospital, Helsinki, Finland

^4^Department of Clinical and Molecular Medicine, Norwegian University of Science and Technology, Trondheim, Norway

^9^Department of Obstetrics and Gynecology, Tampere University Hospital and Faculty of Medicine and Health Technology, Center for Child, Adolescence and Maternal Health, Tampere University, Tampere, Finland

^10^Medical and Clinical Genetics, University of Helsinki and Helsinki University Hospital, Helsinki, Finland

^11^Institute for Molecular Medicine Finland, Helsinki Institute of Life Science, University of Helsinki, Helsinki, Finland

^12^Department of General Practice and Primary Health Care, University of Helsinki and Helsinki University Hospital, Helsinki, Finland

^13^Folkhälsan Research Center, Helsinki, Finland

^14^Department of Obstetrics and Gynecology and Human Potential Translational Research Programme, Yong Loo Lin School of Medicine, National University of Singapore, Singapore

^15^Singapore Institute for Clinical Sciences (SICS), Agency for Science, Technology, and Research, Singapore, Singapore

^16^Institute of Clinical Medicine, Internal Medicine, Turku University Hospital, University of Turku, Turku, Finland

^17^Department of Information Services, Finnish Institute for Health and Welfare, Helsinki, Finland

^18^Academic Primary Health Care Centre, Region Stockholm and Department of Molecular Medicine and Surgery, Karolinska Institute, Stockholm, Sweden

^19^Department of Government Services, Finnish Institute for Health and Welfare, Helsinki, Finland

**Supplemental Table 1. The inter-assay and intra-assay coefficients of variations (CVs).**

|  | **Range of inter-assay controls** | **Inter-assay**  **CV%** | **Intra-assay CV%** |
| --- | --- | --- | --- |
| **Ceramides** |  |  |  |
| Cer(d18:1/16:0) | 0.250–0.324 µmol/L | 8.8% | 5.3% |
| Cer(d18:1/18:0) | 0.069–0.103 µmol/L | 10.7% | 8.2% |
| Cer(d18:1/24:0) | 1.593–2.051 µmol/L | 6.6% | 2.9% |
| Cer(d18:1/24:1) | 1.079–1.276 µmol/L | 5.8% | 2.7% |
| **Traditional lipids** |  |  |  |
| Cholesterol | 4.35–4.99 mmol/L | 3.6% | 2.0% |
| LDL | 2.01–3.12 mmol/L | 6.4% | 4.0% |
| HDL | 0.71–1.02 mmol/L | 5.9% | 2.5% |
| Triglycerides | 1.51–1.99 mmol/L | 4.2% | 2.5% |

Cer, ceramide; HDL, high-density lipoprotein; LDL, low-density lipoprotein

**Supplemental Table 2.** **Odds ratios (ORs) per quartiles for gestational diabetes (GDM) (*n* = 1,998).**

| **Parameter** |  |  |  |  |
| --- | --- | --- | --- | --- |
| **Ceramides** |  |  |  |  |
| **Cer(d18:1/16:0)** | Quartile 1 | Quartile 2 | Quartile 3 | Quartile 4 |
| Subjects | 499 | 500 | 500 | 499 |
| Range, µmol/L | 0.14–0.24 | 0.25–0.28 | 0.29–0.33 | 0.34–0.72 |
| Model 1 | 1.00 | 1.20 (0.93 – 1.54) | 1.18 (0.92 – 1.51) | 1.43 (1.11 – 1.83) |
| Model 2 | 1.00 | 1.01 (0.76 – 1.33) | 1.00 (0.75 – 1.32) | 1.16 (0.87 – 1.55) |
| Model 3 | 1.00 | 1.02 (0.77 – 1.36) | 0.97 (0.72 – 1.29) | 1.09 (0.81 – 1.47) |
| **Cer(d18:1/18:0)** | Quartile 1 | Quartile 2 | Quartile 3 | Quartile 4 |
| Subjects | 499 | 500 | 500 | 499 |
| Range, µmol/L | 0.02–0.06 | 0.07–0.08 | 0.09–0.10 | 0.11–0.33 |
| Model 1 | 1.00 | 1.43 (1.12 – 1.84) | 1.57 (1.22 – 2.01) | 2.40 (1.86 – 3.09) |
| Model 2 | 1.00 | 1.27 (0.96 – 1.67) | 1.11 (0.84 – 1.47) | 1.24 (0.92 – 1.67) |
| Model 3 | 1.00 | 1.22 (0.92 – 1.62) | 1.06 (0.79 – 1.42) | 1.10 (0.80 – 1.50) |
| **Cer(d18:1/24:0)** | Quartile 1 | Quartile 2 | Quartile 3 | Quartile 4 |
| Subjects | 499 | 500 | 500 | 499 |
| Range, µmol/L | 0.64–1.67 | 1.68–1.97 | 1.98–2.33 | 2.34–6.29 |
| Model 1 | 1.00 | 1.43 (1.12 – 1.84) | 1.60 (1.25 – 2.06) | 2.11 (1.64 – 2.72) |
| Model 2 | 1.00 | 1.25 (0.95 – 1.65) | 1.34 (1.01 – 1.78) | 1.59 (1.19 – 2.11) |
| Model 3 | 1.00 | 1.21 (0.90 – 1.61) | 1.32 (0.99 – 1.77) | 1.44 (1.07 – 1.95) |
| **Cer(d18:1/24:1)** | Quartile 1 | Quartile 2 | Quartile 3 | Quartile 4 |
| Subjects | 499 | 500 | 500 | 499 |
| Range, µmol/L | 0.50–1.03 | 1.04–1.20 | 1.21–1.44 | 1.45–4.25 |
| Model 1 | 1.00 | 1.07 (0.84 – 1.38) | 1.55 (1.21 – 1.99) | 2.21 (1.71 – 2.84) |
| Model 2 | 1.00 | 0.89 (0.68 – 1.18) | 1.20 (0.91 – 1.58) | 1.20 (0.89 – 1.60) |
| Model 3 | 1.00 | 0.83 (0.62 – 1.11) | 1.16 (0.86 – 1.54) | 1.05 (0.77 – 1.44) |
| **Cer(d18:1/18:0)/**  **Cer(d18:1/16:0) ratio** | Quartile 1 | Quartile 2 | Quartile 3 | Quartile 4 |
| Subjects | 499 | 500 | 500 | 499 |
| Range | 0.07–0.23 | 0.24–0.28 | 0.29–0.35 | 0.36–1.20 |
| Model 1 | 1.00 | 1.35 (1.06 – 1.74) | 1.52 (1.18 – 1.94) | 2.38 (1.84 – 3.07) |
| Model 2 | 1.00 | 1.20 (0.91 – 1.57) | 1.12 (0.84 – 1.46) | 1.29 (0.96 – 1.73) |
| Model 3 | 1.00 | 1.22 (0.91 – 1.62) | 1.06 (0.79 – 1.42) | 1.19 (0.88 – 1.62) |
| **Traditional lipids** |  |  |  |  |
| **Cholesterol** | Quartile 1 | Quartile 2 | Quartile 3 | Quartile 4 |
| Subjects | 497 | 501 | 502 | 498 |
| Range, mmol/L | 2.44–4.14 | 4.15–4.66 | 4.67–5.20 | 5.21–7.74 |
| Model 1 | 1.00 | 1.39 (1.09 – 1.79) | 1.63 (1.27 – 2.09) | 1.94 (1.51 – 2.49) |
| Model 2 | 1.00 | 1.10 (0.83 – 1.45) | 1.22 (0.92 – 1.62) | 1.42 (1.06 – 1.90) |
| Model 3 | 1.00 | 1.12 (0.84 – 1.50) | 1.13 (0.84 – 1.52) | 1.32 (0.97 – 1.80) |
| **LDL** | Quartile 1 | Quartile 2 | Quartile 3 | Quartile 4 |
| Subjects | 494 | 511 | 499 | 494 |
| Range, mmol/L | 0.72–1.92 | 1.93–2.33 | 2.34–2.82 | 2.83–5.64 |
| Model 1 | 1.00 | 1.77 (1.37 – 2.27) | 2.50 (1.94 – 3.23) | 2.95 (2.28 – 3.82) |
| Model 2 | 1.00 | 1.46 (1.10 – 1.92) | 1.81 (1.36 – 2.41) | 1.87 (1.39 – 2.52) |
| Model 3 | 1.00 | 1.39 (1.04 – 1.85) | 1.75 (1.30 – 2.37) | 1.63 (1.19 – 2.24) |
| **HDL** | Quartile 1 | Quartile 2 | Quartile 3 | Quartile 4 |
| Subjects | 491 | 526 | 489 | 492 |
| Range, mmol/L | 0.38–0.71 | 0.72–0.82 | 0.83–0.94 | 0.95–1.74 |
| Model 1 | 1.00 | 0.94 (0.84 – 1.21) | 0.82 (0.64 – 1.06) | 0.62 (0.48 – 0.79) |
| Model 2 | 1.00 | 0.94 (0.71 – 1.25) | 0.92 (0.69 – 1.23) | 0.75 (0.56 – 1.00) |
| Model 3 | 1.00 | 0.91 (0.68 – 1.23) | 0.91 (0.67 – 1.24) | 0.81 (0.60 – 1.10) |
| **Triglycerides** | Quartile 1 | Quartile 2 | Quartile 3 | Quartile 4 |
| Subjects | 499 | 508 | 495 | 496 |
| Range, mmol/L | 0.50–1.06 | 1.07–1.37 | 1.38–1.81 | 1.82–13.0 |
| Model 1 | 1.00 | 1.33 (1.03 – 1.70) | 1.42 (1.07 – 1.89) | 3.82 (2.93 – 4.97) |
| Model 2 | 1.00 | 1.40 (1.06 – 1.84) | 1.55 (1.16 – 2.06) | 2.39 (1.75 – 3.25) |
| Model 3 | 1.00 | 1.42 (1.07 – 1.89) | 1.59 (1.18 – 2.15) | 2.17 (1.57 – 3.00) |

Cer: ceramide; HDL, high-density lipoprotein; LDL, low-density lipoprotein

Model 1: Linear regression, unadjusted

Model 2: Linear regression adjusted for pre-pregnancy BMI, maternal age, parity (dichotomous variable) and gestational weeks at sampling

Model 3: Linear regression adjusted for Model 2 and education, history of GDM, parental type 2 diabetes and delivery hospital

**Supplemental Figure 1. The directed acyclic graph summarises the hypothetical causality between GDM, ceramides and traditional lipids, and potential confounding variables used in the regression analyses.**

*
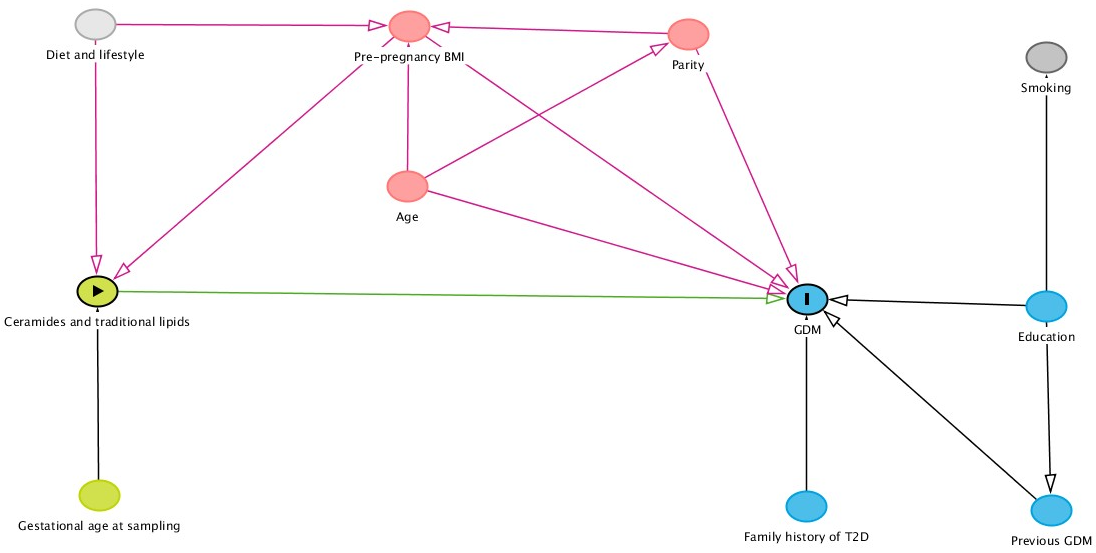
*

The green oval with triangle (ceramides and traditional lipids) represents exposure, the blue (I) oval (GDM) is an outcome, the pink ovals (pre-pregnancy BMI, parity and age) are the precursors of exposure and the outcome (confounders), the clear blue ovals (education, family history of GDM, previous GDM) are the ancestors of the outcome (potential confounders), the green arrow demonstrates the hypothetical causal path, and the pink arrows demonstrate biasing paths. The clear green oval (gestational age at sampling) is a technical variable.

**Supplemental Figure 2. Receiver operating characteristic (ROC) curve and sensitivity and specificity for models including the clinical risk factors (solid curves in panels A, B and C) and, in addition, the traditional lipids (dashed curves panels A,B and C).**

**
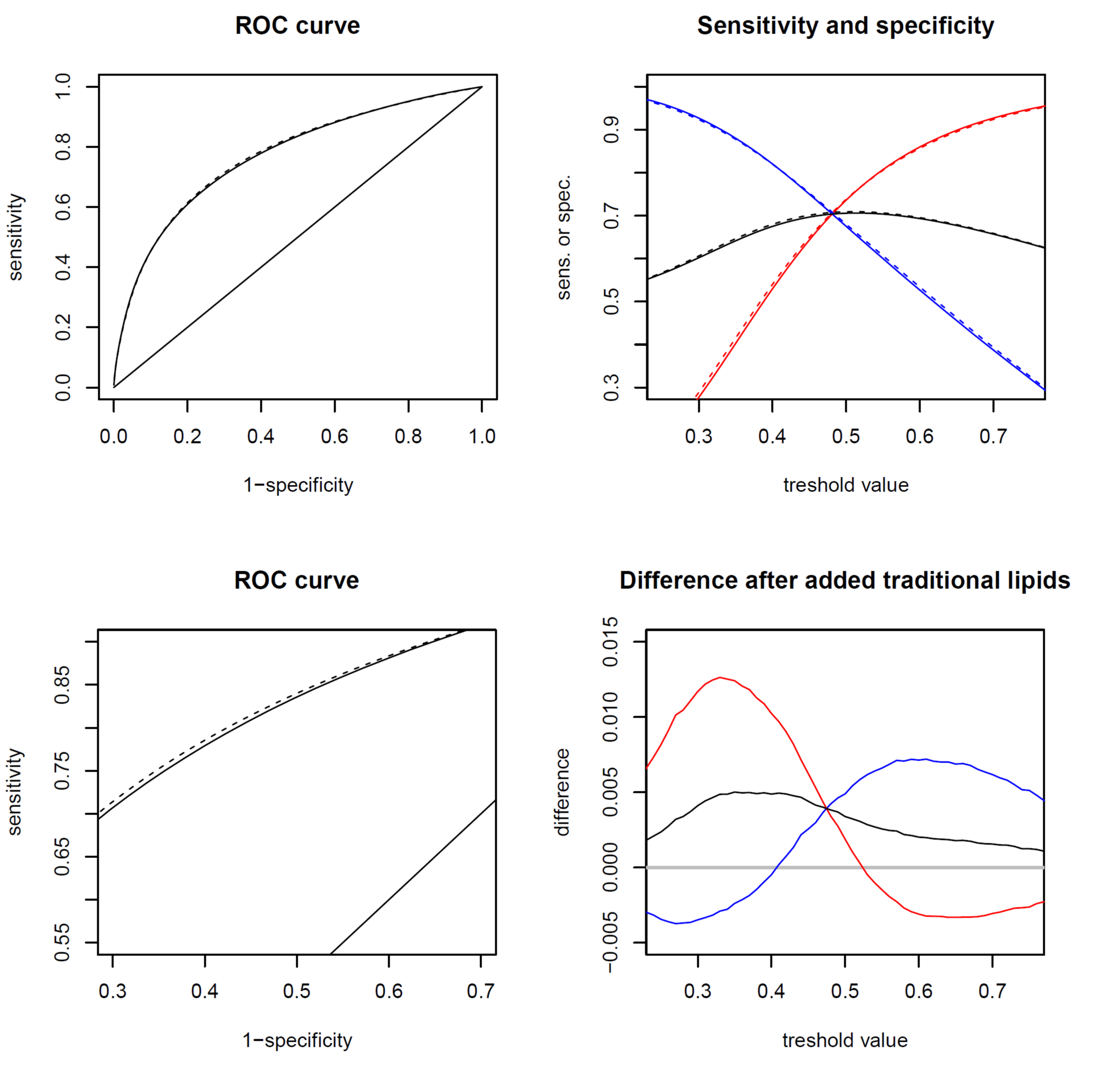
**

Predictions were obtained using a binary LASSO regression model in a repeated cross-validation setup. 10-fold cross validation was repeated 100 times to obtain the out-of-sample predictions. Average performance, measured by sensitivity and specificity, at varying classification threshold values was calculated.

Panels A and C: ROC curves.

Panel B: Sensitivity (blue curve) and specificity (red curve) and their average value (with equal weight, black line) at varying threshold values.

Panel D: Improvement in sensitivity (blue line), specificity (red line) and their average (with equal weight, black line) at varying threshold values after the traditional lipids are added to the model. Positive values indicate improved classification performance.
